# Extracts from the “London Lancet’

**Published:** 1856-01

**Authors:** J. Richardson

**Affiliations:** Cincinnati


Dr. Taylor, Dear Sir:—I find the following case reported in the October number, (1855,) of the "London Lancet," By Harry Dove, M. R. C. S., &c.

Believing that the history of the case, peculiar and extraordinary in its nature, and rendered still more so by the treatment and its singular success, will be acceptable to your readers, I transcribe and submit it to you for publication in the "Register."

Without attempting any speculations upon the exact nature of the sympathies or relations (if any) that may have existed between the teeth in question and those terrible and afflictive manifestations of perverted nervous sensation and muscular action as recorded by the author, we would only suggest that the prompt subsidence of many of the more urgent symptoms and the subsequent gradual improvement in the condition of the patient, point to the removal of the decayed teeth as the prominent, and as we are inclined to think, the *chief* agent effecting the remarkable and almost miraculous results that were ultimately attained in the management of the case. If such be the case, it is an instance worthy of lasting record and remembrance.

We have no means of arriving at any thing like a satisfactory conclusion as to the nature and extent of the relations existing between the teeth, and the nervous system generally in this case, except by deductions predicated upon the results of treatment. The history of the case will not warrant us in the belief that the singular complication of disordered action characterizing the malady in question, had its *origin* in any structural or functional lesion of the dental organs, but it is a subject proper for speculative inquiry, how far this latter condition may have exercised a peculiar and mysterious influence in exaggerating, confirming and perpetuating an abnormal condition, primarily located in some of the nervous centres. We infer, that to a considerable extent, such was the case. There was but one other mode of treatment adopted, and that consisted in conforming the patient forcibly and persistently to certain postures, for no other purpose we imagine, than to overcome confirmed distortions, by inducing relaxation of parts, rendered rigid by long-continued and irregular muscular contractions. As reaching and subduing primary conditions of morbid action therefore, we may, on the principle of exclusion and by fair inference, give precedence to the former treatment as being essentially *curative*, assigning to the latter an agency as *auxiliary* only, to the former. For, as the author assures us, upon the removal of the teeth the "neuralgia of the left side and of the face and head was cured; she was enabled to take more food, and her health was much improved." We hear no more of those fearful convulsions and spasms, of the violent spasmodic coughing, or the habitual ejection of food. And although *all* of the deranged functions were not perfectly restored to their normal condition for many months afterwards, yet this fact does not by any means invalidate the conclusion we have drawn. We regret that the author has not furnished us his views more fully upon the inquiry suggested in the preceding remarks, as it is undoubtedly a legitimate and important one, and essential to an intelligent comprehension of the propriety and *rationale* of the means of cure adopted.

We also extract from the same number of the "Lancet," as containing matter interesting to the profession, two or three brief articles, the-subject matter of which is indicated by their several titles. Truly, &c.,

J. RICHARDSON.

*Cincinnati*, December, 1855.

